# PPDPF Promotes the Progression and acts as an Antiapoptotic Protein in Non-Small Cell Lung Cancer

**DOI:** 10.7150/ijbs.65654

**Published:** 2022-01-01

**Authors:** Mu Yun, Li Yingzi, Gao Jie, Liu Guanxin, Zeng Zimei, Cao Zhen, Li Zhi, Nie Yingjie, Sun Lunquan, Chen Tao, Deng Yuezhen, Zhou Chengzhi

**Affiliations:** 1 Department of Oncology, Xiangya Cancer Center, Xiangya Hospital, Central South University, Changsha 410008, China.; 2 State Key Laboratory of Respiratory Disease, National Clinical Research Center for Respiratory Disease, Guangzhou Institute of Respiratory Health, the First Affiliated Hospital of Guangzhou Medical University, Guangzhou 510120, China.; 3 Key Laboratory of Molecular Radiation Oncology Hunan Province, Changsha 410008, China.; 4 Hunan International Science and Technology Collaboration Base of Precision Medicine for Cancer, Changsha 410008, China.; 5 Center for Molecular Imaging of Central South University, Xiangya Hospital, Changsha 410008, China.; 6 NHC Key Laboratory of Pulmonary Immune-related Diseases, Guizhou Provincial People's Hospital; Guiyang 550000, China.

**Keywords:** PPDPF, NSCLC, BABAM2, MDM2, radioresistance

## Abstract

Resistance to radiotherapy is frequently observed in the clinic and leads to poor prognosis of non-small cell lung cancer (NSCLC). How to overcome resistance to radiotherapy is a challenge in the treatment of NSCLC. In this study, PPDPF was found to be upregulated in NSCLC tissues and cell lines, and its expression negatively correlated with the overall survival of patients with NSCLC. PPDPF promoted the growth, colony formation and invasion of lung cancer cells. Moreover, knockout of PPDPF inhibited tumorigenesis in the KL (Kras^G12D^; LKB1^f/f^) mouse model of lung cancer. Additionally, overexpression of PPDPF led to radioresistance in lung cancer cells, and knockdown of PPDPF sensitized lung cancer cells to radiotherapy. Mechanistically, PPDPF interacted with BABAM2 (an antiapoptotic protein) and blocked its ubiquitination by MDM2, thus stabilizing BABAM2 and promoting the radioresistance of lung cancer cells. Our present study suggested PPDPF as a therapeutic target in NSCLC.

## Introduction

Lung cancer is the second leading cause of cancer-related death worldwide 1. Lung adenocarcinoma (LUAD) and lung squamous carcinoma (LUSC) are the most common subtypes 1-4. Currently, lung cancer is primarily treated with surgery, chemotherapy and radiotherapy 2. Owing to different degrees of resistance to radiotherapy in patients, the effect of radiotherapy decreases, making it clinically imperative to elucidate the mechanism of resistance to radiotherapy 5-7.

Evasion of apoptosis is one of the characteristics of tumor cells. Through evasion of apoptosis, tumor cells promote the recurrence and progression of tumors as well as their resistance to treatment 8-10. Radiotherapy is effective in treating tumors by increasing the number of DNA double-strand breaks (DSBs) or inhibiting the repair of DNA damage 11-14. Radiotherapy-induced DSBs are the most severe type of DNA damage and can be repaired through two main processes, *i.e.*, nonhomologous end joining (NHEJ) and homologous recombination (HR) 15, 16. The carboxyl terminal of H2AX at the breakpoint is rapidly phosphorylated at the serine-139 position (to form γH2AX) in response to a broad range of DNA lesions 17, 18. γH2AX is generally recognized as one of the signs of DSBs, and its dynamic changes reflect the severity and repair of DNA damage 19. The BRISC and BRCA1-A complex member 2 (BABAM2) protein is very conserved 20. It can specifically interact with p55 and Fas to maintain the level of X-linked inhibitor of apoptosis (XIAP) protein to promote apoptosis resistance 21, 22. In addition, the complex composed of BABAM2 and MERIT40 participates in the repair of DNA damage mediated by nuclear assembly and the Breast Cancer Susceptibility Protein-1 (BRCA1) complex 23-25.

PPDPF is the 149^th^ open reading frame (ORF) located on human chromosome 20^26^, ^27^, which was reported to be a key regulator for the development of exocrine pancreas 26, 28. Recently, PPDPF, JUN and HES4 together are found to be a transcriptomic signature that could predict biochemical recurrence with better accuracy in prostate cancer 29, suggesting a role of PPDPF in the treatment response of cancers. However, the detailed function and mechanisms of PPDPF in cancer development remains to be elucidated.

In this study, we investigated the expression pattern of PPDPF in lung cancer, the effect of PPDPF on the resistance of lung cancer to treatment, and attempted to further elucidate the underlying mechanism.

## Results

### The expression of PPDPF is upregulated in NSCLC

**Figure 1:**
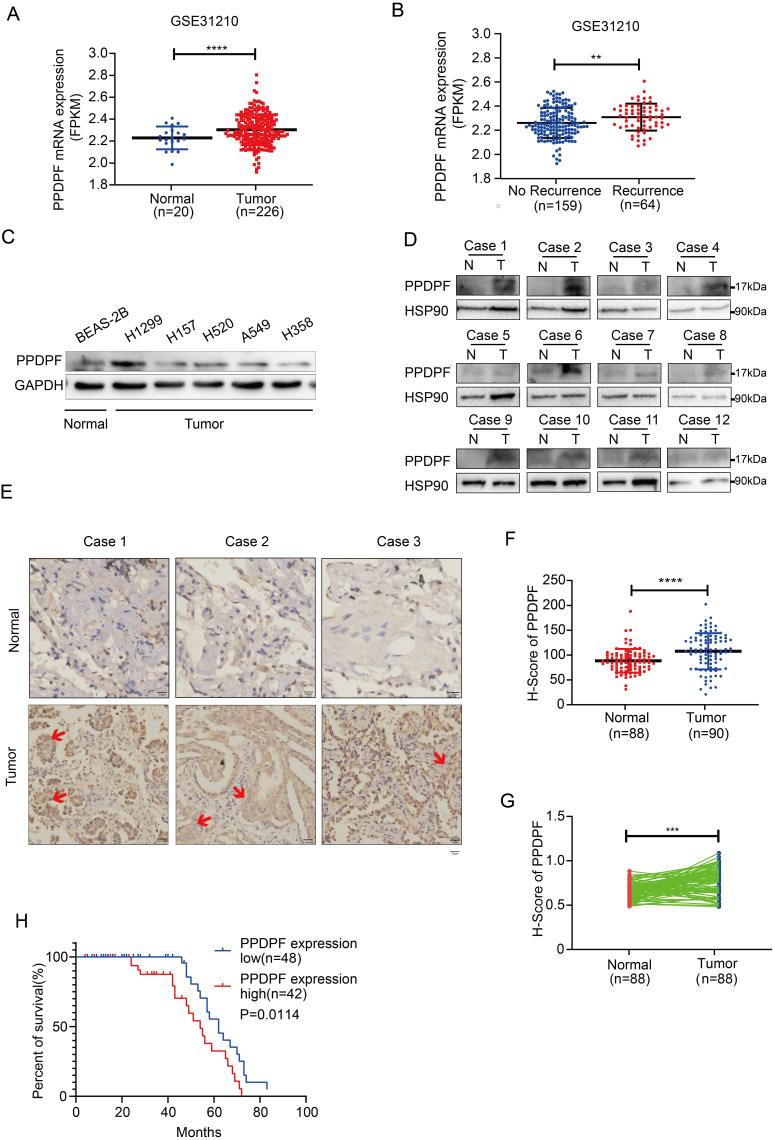
** Expression of PPDPF was elevated in lung cancer. (A-B)** The GSE31210 dataset was analyzed the expression of PPDPF in lung cancer. PPDPF was upregulated in lung cancer. (A). Moreover, PPDPF mRNA levels were higher in NSCLC with recurrence than in NSCLC without recurrence. (B) **, *P*<0.01. ****, *P*<0.0001, Student's *t* test. **(C)** Western blotting was used to detect the expression of PPDPF protein levels in NSCLC cells (normal bronchiolar cells: BEAS-2B; NSCLC cells: A549, H1299, H157, H520 and H358). GAPDH was used as a loading control. **(D)** Western blotting was performed to detect the expression of PPDPF in 12 samples of NSCLC tissues (T) and matched adjacent tissues (N). HSP90 was used as the internal reference. **(E-G)** The expression of PPDPF in the NSCLC tissue array was detected by IHC and scored. The tissue array contained 88 samples of normal tissues and 90 samples of NSCLC tissues. Representative images of three sets of samples (normal and tumor tissues) are shown and positive signal of PPDPF stain in tumor tissue was indicated with red arrow. Scale bar: 100 µm. (E). Immunohistochemical staining and scoring were performed as described in the “Materials and Methods” section. The scores of the normal tissues and the tumor tissues were compared (F). The scores of the normal tissues and the paired tumor tissues were analyzed (G). **(H)** Kaplan-Meier analysis was used to analyze the relationship between the expression of PPDPF with the overall survival of NSCLC patients. Survival curves were constructed using the Kaplan-Meier method and were analyzed by the log-rank test (*, *P* < 0.05).

We first analyzed the GSE31210 lung cancer dataset, and the results suggested that the expression of PPDPF was upregulated in the lung cancer tissues (Figure 1A). Moreover, the expression of PPDPF was higher in tumor tissues in recurrent cases than that in nonrecurrent cases (Figure 1B). In addition, we examined the expression pattern of PPDPF in lung cancer samples and cell lines. Western blotting was performed to measure the protein level of PPDPF in lung cancer cells. The results showed that the protein level of PPDPF was relatively high in the lung cancer cell lines (Figure 1C). In addition, the protein level of PPDPF was examined in the 12 adjacent tissues and paired tumor tissues derived from the lung cancer patients (Table S1). It was found that the protein level of PPDPF was significantly elevated in lung cancer tissues (10/12) (Figure 1D). To further confirm these observations, we examined the protein level of PPDPF in a lung cancer tissue array (containing 90 lung cancer tissues and 88 paired adjacent tissues, Table S2). Consistent with the above findings, the results from the immunohistochemistry (IHC) showed that the protein level of PPDPF was significantly increased in the cancer tissues (Figure 1E-G). Furthermore, the protein level of PPDPF was negatively correlated with the overall survival (Figure 1H). These findings indicate that PPDPF may promote the progression of lung cancer.

### PPDPF promotes the growth, colony formation and invasion of lung cancer cells

**Figure 2:**
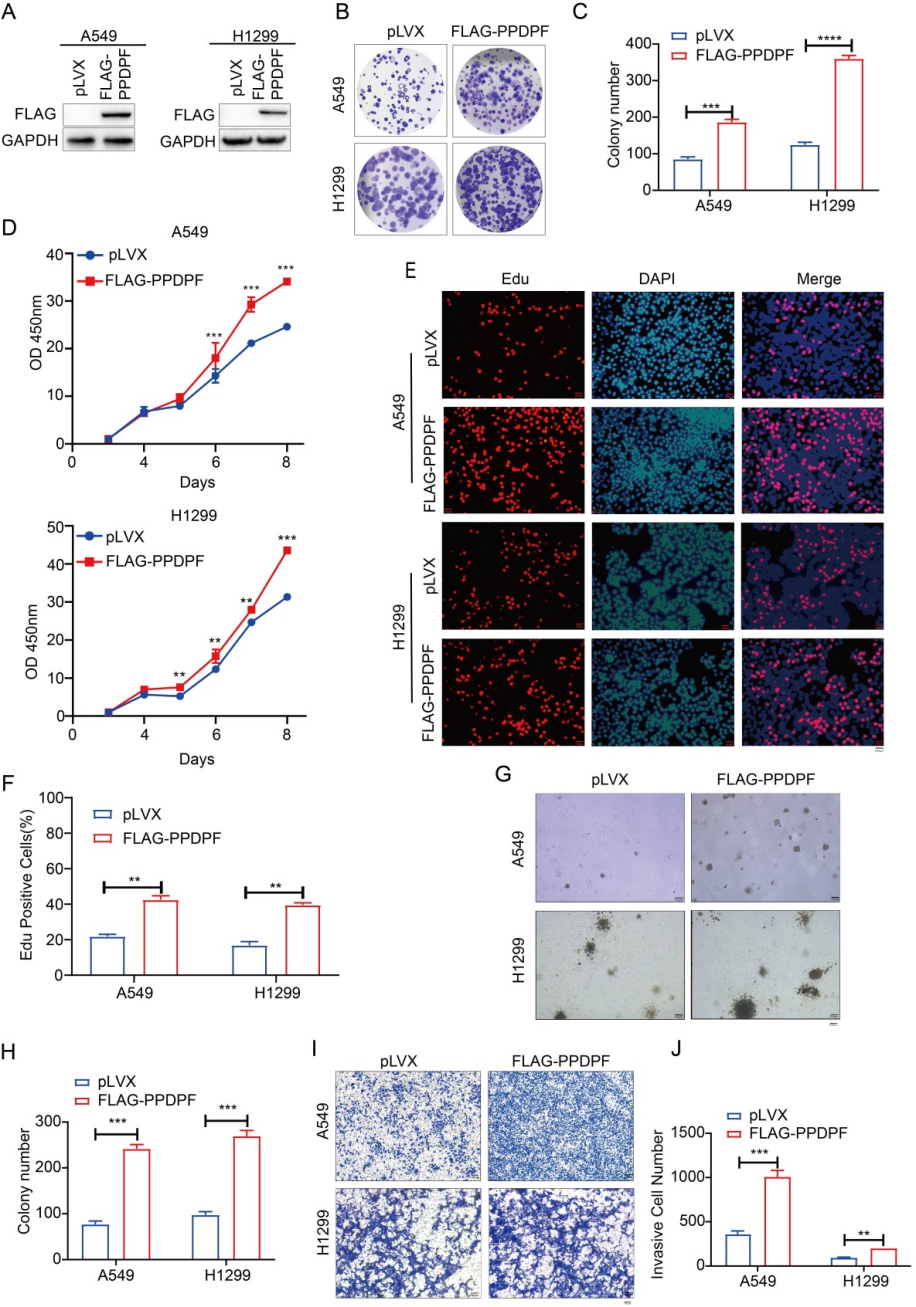
** PPDPF promoted the growth, proliferation and invasion of lung cancer cells. (A)** Forced expression of PPDPF in A549 and H1299 cells. After being infected with lentivirus expressing pLVX (control) and PPDPF, A549 and H1299 cells were screened for 7 days with puromycin. The resistant cells were pooled, and the expression level of PPDPF was determined by western blotting. **(B-C)** A crystal violet assay was performed to evaluate the effects of PPDPF overexpression on the growth of A549 and H1299 cells. Data are presented as mean ±SD from n=3, *, *P*<0.05; **, *P*<0.01; ***, *P*<0.001, Student's *t* test. **(D)** A CCK-8 assay was performed to evaluate the effects of PPDPF overexpression on the growth of A549 and H1299 cells. Data are presented as mean ±SD from n=3, *, *P*<0.05; **, *P*<0.01; ***, *P*<0.001, Student's *t* test. **(E-F)** An EdU incorporation assay was performed to evaluate the effects of PPDPF overexpression on the proliferation of A549 and H1299 cells. Scale bar: 100 µm. Data are presented as mean ±SD from n=3, *, *P*<0.05; **, *P*<0.01; ***, *P*<0.001, Student's *t* test. **(G-H)** A soft agar colony formation assay was performed to evaluate the effects of PPDPF overexpression on the anchorage-independent growth of A549 and H1299 cells. Scale bar: 100 µm. Data are presented as mean ± SD from n=4. **(I-J)** A Transwell assay was performed to evaluate the effects of PPDPF overexpression on the invasion of A549 and H1299 cells. Scale bar: 100 µm. Data are presented as mean ±SD from n=3, *, *P*<0.05; **, *P*<0.01; ***, *P*<0.001, Student's *t* test.

To study the effect of PPDPF on the progression of lung cancer, Flag-tagged PPDPF (Flag-PPDPF) was overexpressed in two lung cancer cell lines (A549 and H1299) (Figure 2A). In the colony formation assay and CCK-8 assay, overexpression of PPDPF promoted the growth of lung cancer cells in liquid culture (Figure 2B-D). Moreover, the EdU incorporation assay showed that forced expression of PPDPF promoted the proliferation of lung cancer cells (Figure 2E-F). In addition, we assessed the anchorage-independent growth of A549 and H1299 cells after overexpression of PPDPF using a soft agar assay. PPDPF enhanced both the anchorage-independent growth (Figure 2G-H) and the invasion (Figure 2I-J) of lung cancer cells.

**Figure 3:**
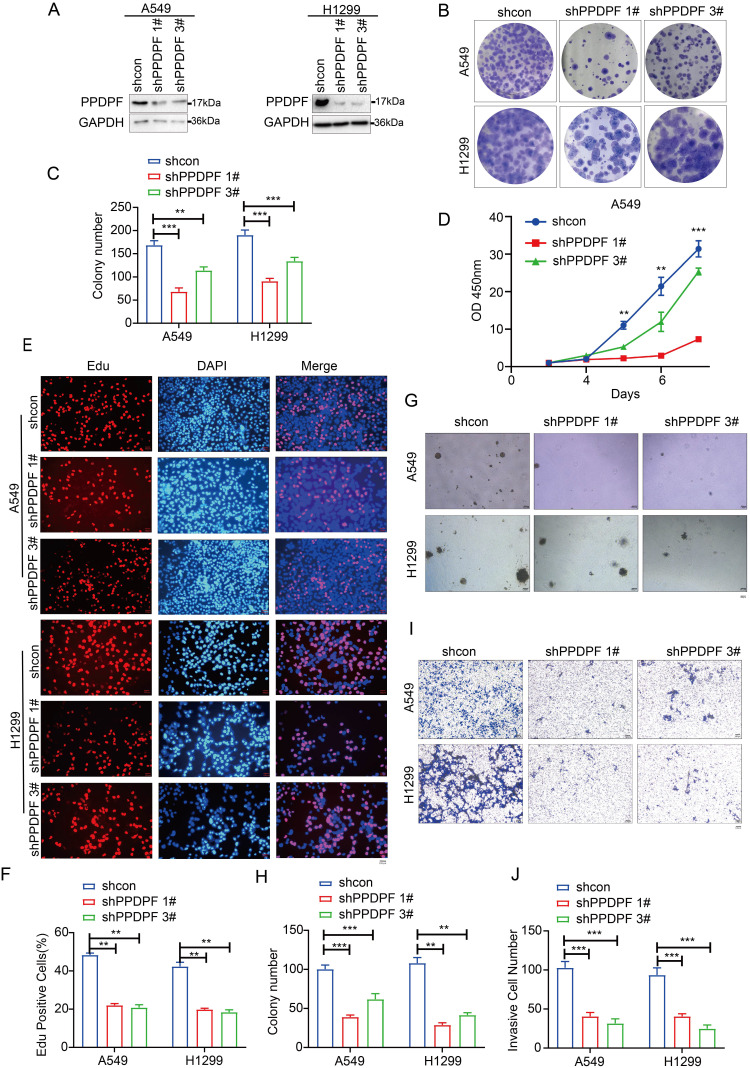
** Knocking down PPDPF inhibited the growth, colony formation and invasion of lung cancer cells. (A)** Knockdown of PPDPF expression in A549 and H1299 cells. After being infected with viral vectors expressing shcon and shPPDPF, A549 and H1299 cells were screened for 7 days with puromycin. The resistant cells were pooled, and the expression of PPDPF was determined by western blotting. **(B-C)** A crystal violet assay was performed to evaluate the effects of PPDPF knockdown on the growth of A549 and H1299 cells. Data are presented as mean ±SD from n=3, *, *P*<0.05; **, *P*<0.01; ***, *P*<0.001, Student's *t* test. **(D)** A CCK-8 assay was performed to evaluate the effects of PPDPF knockdown on the growth of A549 and H1299 cells. Data are presented as mean ±SD from n=3, *, *P*<0.05; **, *P*<0.01; ***, *P*<0.001, Student's *t* test. **(E-F)** An EdU incorporation assay was performed to evaluate the effects of PPDPF knockdown on the proliferation of A549 and H1299 cells. The positive cells were counted for statistical analysis. Scale bar: 100 µm. Data are presented as mean ±SD from n=3, *, *P*<0.05; **, *P*<0.01; ***, *P*<0.001, Student's *t* test. **(G-H)** A soft agar colony formation assay was performed to evaluate the effects of PPDPF knockdown on the anchorage-independent growth of A549 and H1299 cells. The colonies were counted for statistical analysis. Scale bar: 100 µm. Data are presented as mean ± SD from n=4. **(I-J)** A Transwell assay was performed to evaluate the effects of PPDPF knockdown on the invasion of A549 and H1299 cells. The invasive cells were counted for statistical analysis. Scale bar: 100 µm. Data are presented as mean ±SD from n=3, *, *P*<0.05; **, *P*<0.01; ***, *P*<0.001, Student's *t* test.

We next examined the roles of endogenously expressed PPDPF. The results of the crystal violet assay and CCK-8 assay showed that knockdown of PPDPF inhibited the growth of lung cancer cells in liquid medium (Figure 3A-D). Consistent with these results, in the EdU incorporation assay, knockdown of PPDPF inhibited the proliferation of lung cancer cells (Figure 3E-F). Moreover, downregulation of PPDPF inhibited the anchorage-independent growth of lung cancer cells (Figure 3G-H) and weakened the invasion ability of lung cancer cells (Figure 3I-J). These results indicate that PPDPF promotes the progression of lung cancer.

### PPDPF stabilizes BABAM2

**Figure 4:**
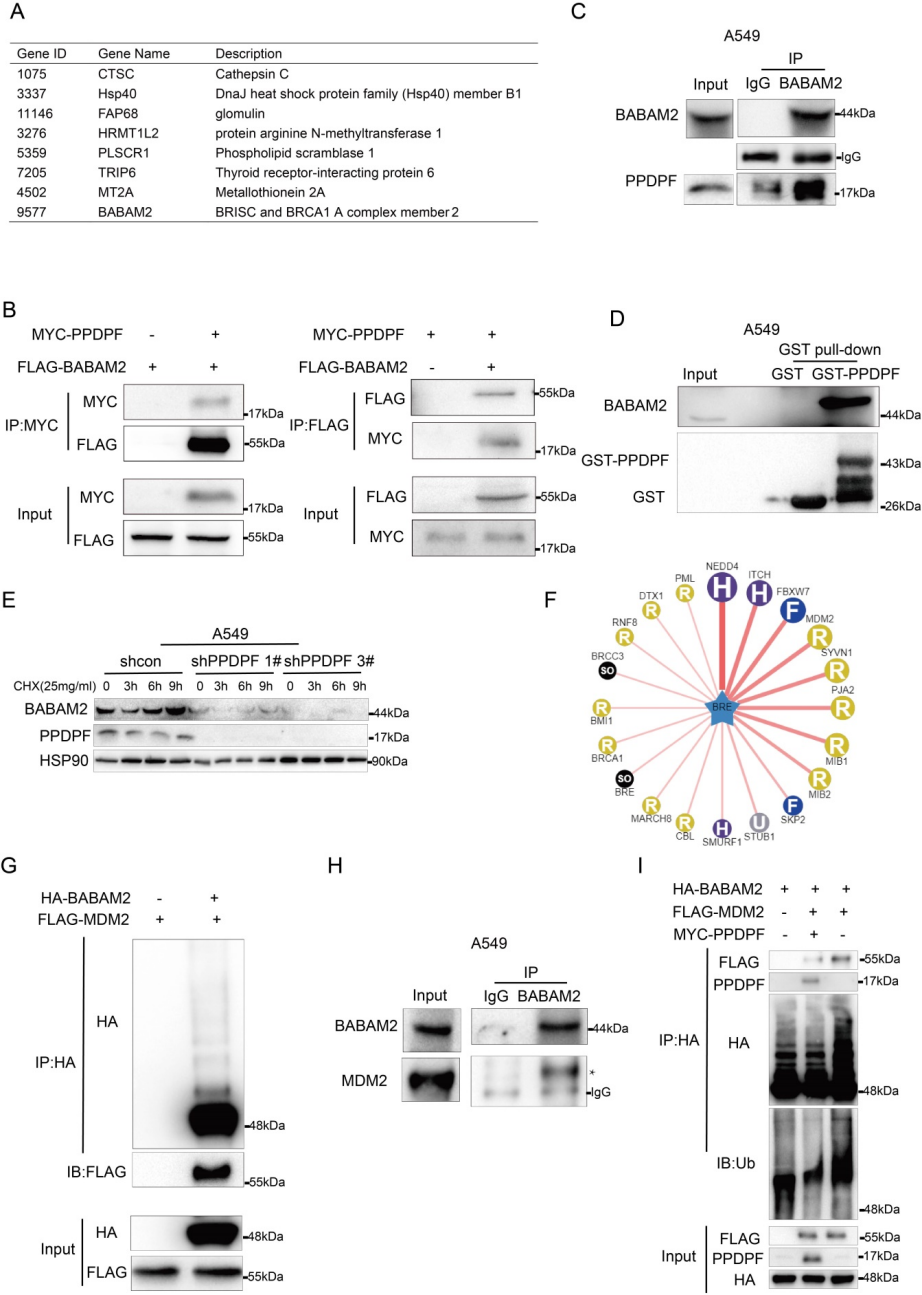
** PPDPF interacted with BABAM2. (A)** The list of the candidate PPDPF binding proteins. **(B)** The interaction between exogenously expressed MYC-PPDPF and Flag-BABAM2 was detected by Co-IP. MYC-PPDPF and Flag-BABAM2 expression plasmids were transfected into HEK293T cells. Forty-eight hours later, the cells were collected for Co-IP. **(C)** The interaction between endogenously expressed PPDPF and BABAM2 was detected by Co-IP in A549 cells. **(D)** A GST pulldown assay was used to examine the binding between endogenously expressed BABAM2 and the fusion protein GST-PPDPF in A549 cells. **(E)** PPDPF knockdown shortened the protein half-life of BABAM2. A549 shcon cells or cells with PPDPF knockdown were treated with 25 mg/ml CHX for the indicated time periods, followed by IB. **(F)** Network view of predicted E3 ligases of BABAM2 in UbiBrowser web services. In network view, the central node is the queried substrate, and the surrounding nodes are the predicted E3 ligases. The width of the edge reflects the confidence of the interaction. **(G)** The interaction between exogenously expressed HA-BABAM2 and Flag-MDM2 was detected by Co-IP. **(H)** An immunoprecipitation assay was used to examine the interaction between endogenous MDM2 and BABAM2 in A549 cells. **(I)** The effects of PPDPF on the ubiquitination of BABAM2 by MDM2 were examined. HEK293T cells were transfected with the indicated plasmids for 48 h and then treated with 10 mM MG132 for 8 h, followed by IP with HA beads and then IB with the indicated Abs.

To elucidate how PPDPF functioned in lung cancer, we used yeast two-hybrid assay to screen PPDPF-binding partners. BABAM2 was a candidate (Figure 4A). In HEK293T cells, an interaction between exogenously expressed PPDPF and BABAM2 was observed (Figure 4B). Furthermore, endogenously expressed PPDPF and BABAM2 formed a complex in lung cancer cells (Figure 4C). In the GST pulldown assay, the fusion protein GST-PPDPF bound to endogenously expressed BABAM2 (Figure 4D). Therefore, BABAM2 might mediate the biological functions of PPDPF. We next determined whether PPDPF regulated the turnover of BABAM2. Using cycloheximide (CHX) to block new protein synthesis, we found that PPDPF knockdown significantly shortened the protein half-life of endogenous BABAM2 in A549 cells (Figure 4E), suggesting that knockdown of PPDPF promoted the degradation of BABAM2. To identify the potential E3 ligases for BABAM2, we queried BABAM2 as substrate in the web tool of UbiBrowser 30 (Figure 4F). The 45 predicted E3 ligases were presented in Table S3. The Top 4 E3 ligases ranked in the table were NEDD4, ITCH, FBXW7 and MDM2, among which MDM2 had been validated to link to radioresistance in human lung cancer cell lines 31, 32. The interaction between MDM2 and BABAM2 were examined using Co-IP assay, and MDM2 proto-oncogene (MDM2) was found to interact with BABAM2 (Figure 4G). BABAM2 formed a complex with endogenously expressed MDM2 in lung cancer cells (Figure 4H). Moreover, MDM2 increased the ubiquitination of BABAM2, which was suppressed by PPDPF (Figure 4I). Together, these results suggest that PPDPF stabilized BABAM2.

### Overexpression of PPDPF contributes to radioresistance in lung cancer cells

**Figure 5:**
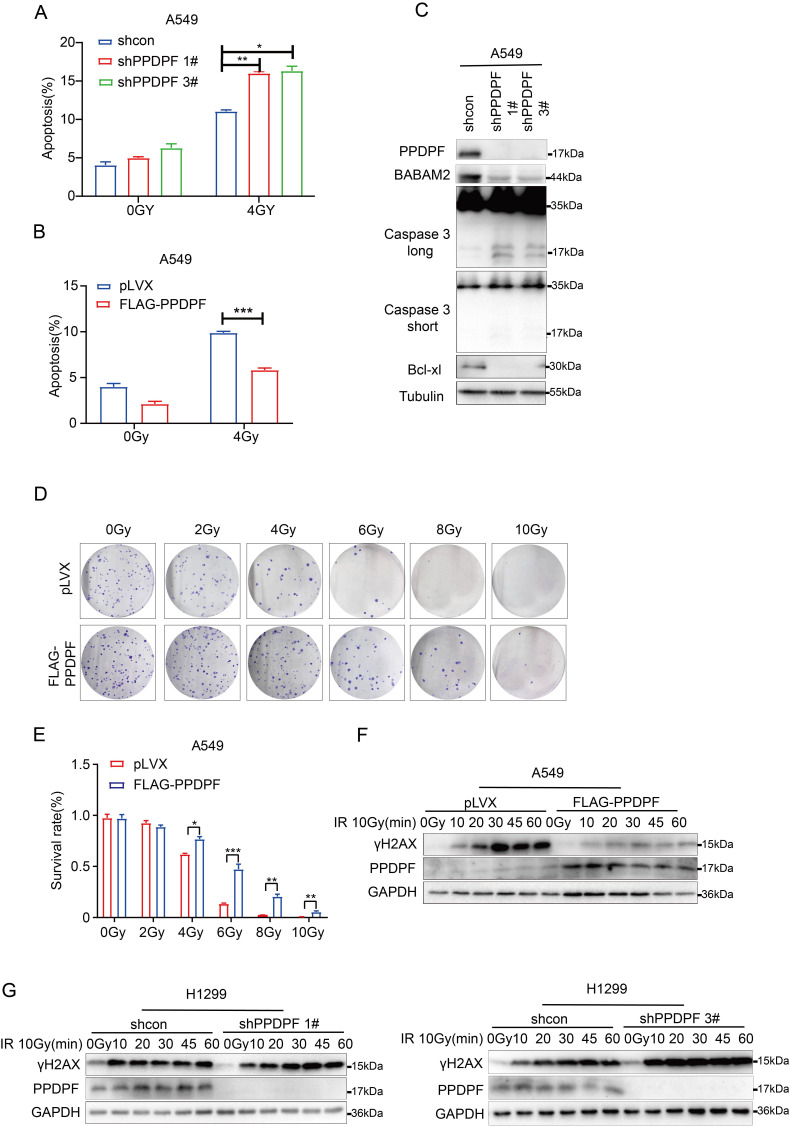
** Overexpression of PPDPF led to radioresistance. (A)** PI-Annexin V staining was performed to examine the apoptosis of A549 cells with PPDPF knockdown. Cells were treated with radiation at a dose of 4 Gy. Twenty-four hours later, the cells were collected, and PI-Annexin V staining was performed for statistical analysis. Data are presented as mean ±SD from n=3, *, *P*<0.05; **, *P*<0.01; ***, *P*<0.001, Student's *t* test. **(B)** PI-Annexin V staining was performed to examine the apoptosis of A549 cells overexpressing PPDPF. Cells were treated with radiation at a dose of 4 Gy. Twenty-four hours later, the cells were collected, and PI-Annexin V staining was performed for statistical analysis. Data are presented as mean ±SD from n=3, *, *P*<0.05; **, *P*<0.01; ***, *P*<0.001, Student's *t* test. **(C)** The protein levels of cleaved Caspase 3, BABAM2 and Bcl-xl in A549 cells with PPDPF knockdown were examined by western blot analysis. **(D-E)** The effects of PPDPF overexpression on the survival of A549 cells were examined with colony formation assay. Equal numbers of cells were seeded in a 6-well plate. Twenty-four hours later, the cells were treated with radiation at the indicated dose. Fourteen days later, the colonies were stained with crystal violet and counted for statistical analysis. Data are presented as mean ±SD from n=3, *, *P*<0.05; **, *P*<0.01; ***, *P*<0.001, Student's *t* test. **(F)** The effects of PPDPF overexpression on the level of γH2AX in A549 cells were examined by western blotting. Cells were treated with radiation at a dose of 10Gy and collected at different post-radiation time points (10 min, 20 min, 30 min, 45 min and 60 min). **(G)** The effects of PPDPF knockdown on the level of γH2AX in H1299 cells were examined by western blotting. Cells were treated with radiation at a dose of 10Gy and collected at different post-radiation time points (10 min, 20 min, 30 min, 45 min and 60 min).

Furthermore, the effect of PPDPF on the resistance to radiotherapy was investigated. Knockdown of PPDPF promoted radiotherapy-induced apoptosis of lung cancer cells (Figure 5A, Figure S1A), and overexpression of PPDPF inhibited radiotherapy-induced apoptosis (Figure 5B, Figure S1B). Consistent with these results, the expression of cleaved Caspase 3 was enhanced, while the protein levels of Bcl-xl and BABAM2 were decreased after knockdown of PPDPF expression (Figure 5C). The colony formation assay results also showed that lung cancer cells overexpressing PPDPF had a higher survival rate after irradiation (Figure 5D-E). In addition, a decreased protein level of γH2AX was observed in lung cancer cells overexpressing PPDPF upon irradiation and the restore time to the basal level was shorter (Figure 5F). Meanwhile, an increased protein level of γH2AX was found in lung cancer cells with PPDPF knockdown upon irradiation and the restore time to the basal level was longer (Figure 5G). These observations indicate that PPDPF promotes DNA damage repair and radioresistance.

### PPDPF promotes the resistance of lung cancer cells to radiotherapy via BABAM2

**Figure 6:**
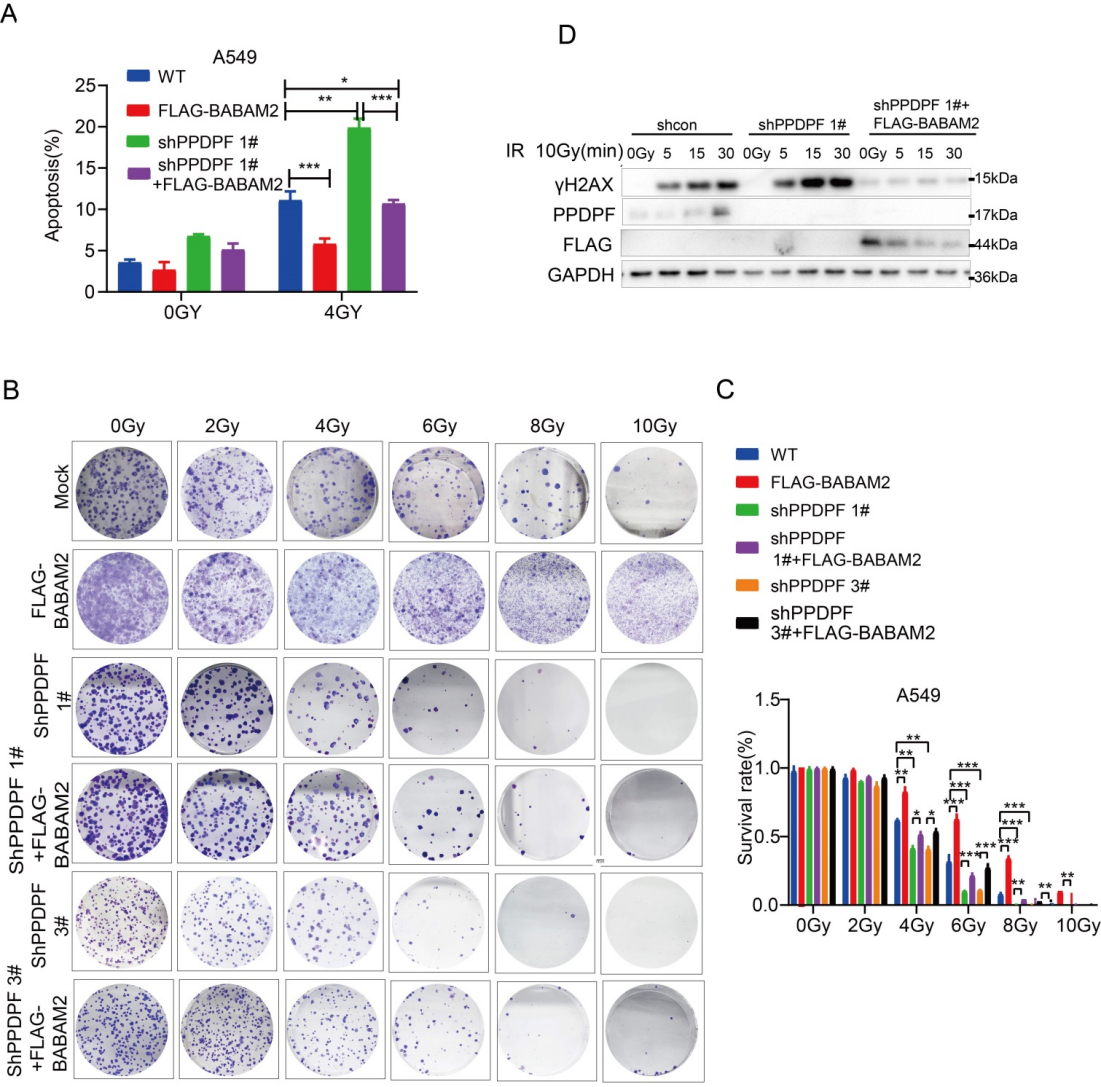
** PPDPF enhanced radioresistance via BABAM2. (A)** PI-Annexin staining was performed to examine the rescue effects of BABAM2 overexpression on apoptosis induced by PPDPF knockdown. Cells were treated with radiation. Twenty-four hours later, apoptosis was evaluated, and statistical analysis was performed. Data are presented as mean ±SD from n=3, *, *P*<0.05; **, *P*<0.01; ***, *P*<0.001, Student's *t* test. **(B-C)** A colony formation assay was performed to examine the rescue effects of BABAM2 overexpression on the survival of A549 cells with PPDPF knockdown. Cells were plated in a 6-well plate and treated with radiation at the indicated dose. Fourteen days later, the colonies were stained with crystal violet, and statistical analysis was performed. Data are presented as mean ±SD from n=3, *, *P*<0.05; **, *P*<0.01; ***, *P*<0.001, Student's *t* test. **(D)** Western blot analysis was performed to examine the rescue effects of BABAM2 overexpression on the level of γH2AX in A549 cells with PPDPF knockdown. Cells were plated in a 6-well plate and treated with radiation. Then, the cells were collected at different post-radiation time points (5 min, 15 min, 30 min), and the protein level of γH2AX was examined.

To verify whether PPDPF promoted radioresistance via BABAM2, irradiation was performed after BABAM2 was overexpressed in lung cancer cells with PPDPF knockdown. The results showed that overexpression of BABAM2 inhibited apoptosis caused by PPDPF knockdown (Figure 6A, Figure S1C). It was observed in the colony formation assay that restoration of BABAM2 expression ameliorated PPDPF knockdown-induced inhibition of cell survival after radiotherapy (Figure 6B-C). Consistent with these results, the expression of γH2AX recovered to the baseline level quickly after the restoration of BABAM2 expression in cells with PPDPF knockdown (Figure 6D), indicating that PPDPF promotes the radioresistance of lung cancer cells via BABAM2.

### Downregulation of PPDPF inhibits the tumorigenesis of lung cancer cells *in vivo*

**Figure 7:**
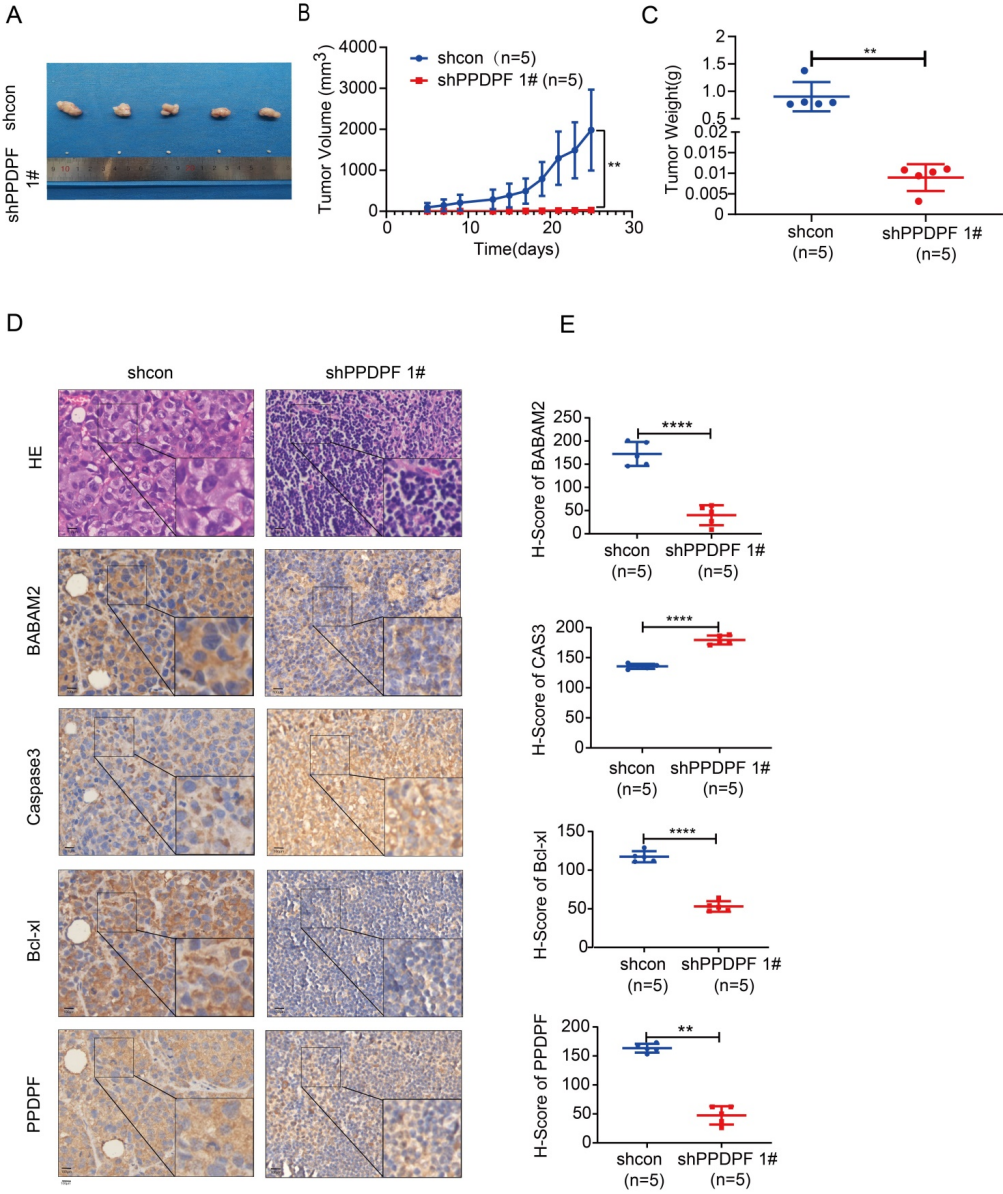
** Down-regulation of PPDPF inhibited tumorigenesis of lung cancer cells *in vivo*. (A-C)** The xenografts formed by H1299 cells. Image of xenografts formed from H1299 control cells and H1299 cells with PPDPF knockdown are shown in (A). The tumor growth curve is shown in (B). The tumor weights are shown in (C). **, *P <* 0.01, Student's *t* test. Details are described in the “Materials and Methods” section. **(D-E)** IHC was performed to examine the protein levels of BABAM2, Caspase3, and Bcl-xl in the xenografts. Scale bar: 100 µm. (D). Immunostaining was scored, and the results of statistical analysis (E). **, *P <* 0.01, ****, *P* < 0.0001, Student's *t* test.

Next, the effect of PPDPF expression on the tumorigenicity of lung cancer cells was explored. Knockdown of PPDPF inhibited subcutaneous tumorigenesis of H1299 cells in nude mice (Figure 7A). Tumors that formed from cells with PPDPF knockdown grew slowly, and were smaller in size and lighter in weight than those formed from control cells (Figure 7A-C). In the xenografts formed from cells with PPDPF knockdown, the IHC results showed that the expression of BABAM2 and Bcl-xl was downregulated and that cleaved Caspase3 was upregulated (Figure 7D-E), further demonstrating that downregulation of PPDPF inhibited the tumorigenesis of lung cancer cells *in vivo*.

### Knockout of PPDPF inhibits the tumorigenesis *de novo*

**Figure 8:**
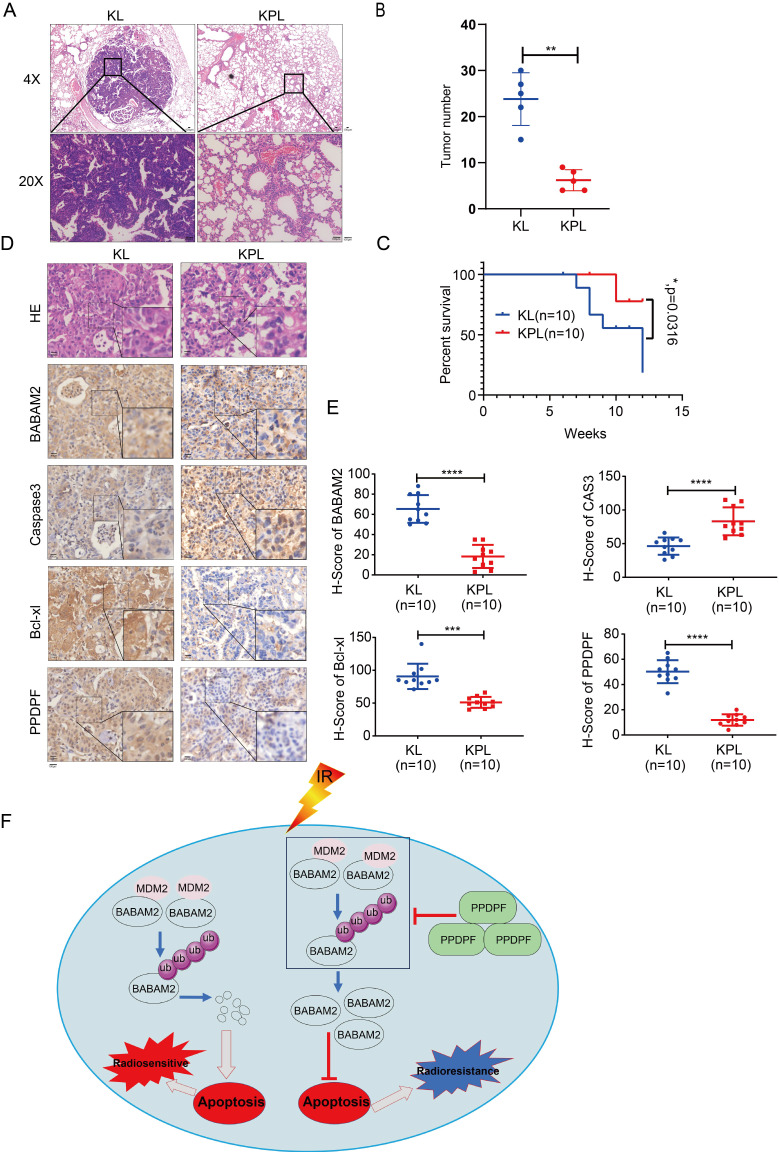
** Knockout of PPDPF inhibited the tumorigenesis *de novo*. (A)** HE staining was performed to examine the tumors in the lungs of KL and KPL mice. Scale bar: 100 µm. KL (Kras^G12D^;LKB1^f/f^) mice were crossed with PPDPF^f/f^ mice to generate KPL (Kras^G12D^;PPDPF^f/f^;LKB1^f/f^) mice. Eight-week-old KL and KPL mice were administered Ad-Cre virus (10^9^ pfu per mouse). Twelve weeks later, the mice were sacrificed, and the lungs were collected for HE staining. **(B)** The statistical analysis of the tumor number. **, *P <* 0.01, Student's *t* test. **(C)**The survival curves of KP and KPL mice. Eight-week-old KL and KPL mice were administered Ad-Cre virus (10^9^ pfu per mouse). The survival of the mice was constructed using the Kaplan-Meier method and were analyzed by the log-rank test (*, *P* < 0.05). **(D-E)** IHC was performed to examine the protein levels of BABAM2, Caspase3, and Bcl-XL in the tumors of KL and KPL mice. Scale bar: 100 µm. (D). Immunostaining was scored, and statistical analysis was performed (E). ***, *P <* 0.001, ****, *P* < 0.0001, Student's* t* test. **(F)** The work model of PPDPF. PPDPF regulates apoptosis via stabilizing BABM2 to induce radioresistance of NSCLC. In the absent of PPDPF, MDM2 binds to BABAM2 and promotes BABAM2 degradation, which in turn promotes apoptosis, thus contributing to the radiosensitivity of NSCLC. In the presence of PPDPF, the ubiquitination of BABAM2 is disrupted and BABAM2 is stabilized, which enhances DNA repair and inhibits apoptosis. As a result, PPDPF confers radioresistance. Arrows represent promotion events, and blunt arrows indicate suppression events.

To further explore the roles of PPDPF in the tumorigenesis, we knocked out PPDPF in the KL (Kras^G12D^; LKB^f/f^) mouse model. Knockout of PPDPF expression in the KL mouse model of lung cancer inhibited tumorigenesis *de novo* (Figure 8A-B), improving the survival of the mice (Figure 8C). The IHC results showed that the expression of BABAM2 and Bcl-xl was downregulated and that cleaved Caspase3 was upregulated after PPDPF was knocked out (Figure 8D-E). In summary, our results demonstrate that deletion of PPDPF inhibited the tumorigenesis of NSCLC *de novo*.

## Discussion

Data in the GEPIA database show that PPDPF is highly expressed in various cancers, including HCC, colorectal cancer, prostate cancer, etc. 33, 34. It was discovered in this study that the expression of PPDPF was upregulated in clinical specimens of lung cancer and that the PPDPF expression level was significantly correlated with the prognosis of patients. PPDPF promoted the growth, invasion and tumorigenic ability of lung cancer cells. In the molecular mechanistic study, PPDPF was found to inhibit apoptosis via stabilizing BABAM2 and to enhance the radioresistance of lung cancer cells (Figure 8F). These findings reveal the important role of PPDPF in the recurrence and progression of lung cancer, suggesting that it is very likely to be a potential target for improving the efficiency of radiotherapy.

A crucial finding in this study was that PPDPF inhibits the apoptotic pathway via stabilizing BABAM2, making lung cancer cells resistant to radiotherapy. Radiotherapy is currently one of the most important approached for the treatment of lung cancer. Current studies show that the activity of the apoptotic pathway and DNA repair pathway is closely related to the effect of radiotherapy 35-38. BABAM2 can cooperate with RAD51, BRCA1and BRCA2 to form BRCA1-BRCA2-containing complex (BRCC) 24, 39-41, a holoenzyme complex containing four subunits. Recent studies suggested that BRCA1 and BRCA2 contribute to DNA repair, transcriptional regulation in response to DNA damage and maintenance of chromosomal stability 41, 42. BRCC, with E3 ubiquitin ligase activity, can repair double-strand breaks in DNA. By binding to Rap80, this complex is recruited to the DNA damage site. In addition, BABAM2 can bind to Fas and TNF-R1 to prevent downstream signaling and inhibit the activation of the mitochondrial apoptotic pathway 38, 43-47. BABAM2 can also exert an antiapoptotic effect by regulating the expression of XIAP 22, 36. It was discovered in this study that BABAM2 mediates the functions of PPDPF in anti-apoptosis and radioresistance.

The present study has found that PPDPF interacted with BABAM2, and PPDPF stabilized BABAM2 by blocking its ubiquitination through MDM2 (Figure 8F). In future studies, determining the therapeutic effects of MDM2 inhibitor will be of great importance for overcoming resistance to radiotherapy.

In summary, this study revealed that the expression of PPDPF is upregulated in lung cancer and inhibits the degradation of BABAM2, thereby enhancing the resistance of lung cancer cells to radiotherapy. These findings suggested that PPDPF is likely to be a therapeutic target for lung cancer.

## Materials and Methods

### Cell culture and transfection

Lung cancer cell lines (A549, H1299, H157, H358 and H520) and a normal bronchial epithelial cell line (Beas-2B) were obtained from the Cell Bank of the Chinese Academy of Sciences (Shanghai, China). All of the lung cancer cell lines were maintained in RPMI-1640 medium (11875-135; Gibco), while Beas-2B cells were cultured in DMEM (11965-084; Gibco). Both media were supplemented with 10% fetal bovine serum (FBS) as well as penicillin and streptomycin (15140122; Gibco). All cell lines were maintained at 37 °C in 5% CO_2_. All cell lines were authenticated.

Cells were transfected with Lipofectamine 8000 (Beyotime) according to the manufacturer's instructions. Stable cell lines were selected by treatment with 2 µg/mL puromycin (Sangon) for 10 days. The resistant cells were pooled, and western blotting was carried out to examine the expression of exogenous PPDPF.

### Clinical samples

Clinical lung cancer samples were obtained from Xiangya Hospital of Central South University. The pathology was determined by the pathologist. The samples were collected after all patients signed informed consent forms. This study was approved by the Institute Research Medical Ethics Committee of Central South University.

### RNA interference

ShRNA targeting PPDPF was designed using software provided by Qiagen (Valencia, CA, USA) and cloned into the pLKO.1-TRC vector. The shRNA target sequences were as follows: shPPDPF #1 (pLKO.1): 5'-CCGGTCCTGACCTGAGCGGTTACCACTCGAGTGGTAACCGCTCAGGTCAGGATTTTT-3'; shPPDPF #3 (pLKO.1): 5'-CCGGGGGTTCCACTTCCAGCAACACTCGAGTGTTGCTGGAAGTGGAACCCATTTTT-3'.

### Western blotting

Lung cancer cell lines were lysed in RIPA buffer containing proteinase (Bimake, B14001) and phosphatase inhibitors (Bimake, B15001), and protein concentrations were quantified using BCA reagent (Thermo Fisher, 23225). Then, the prepared protein was separated via SDS-PAGE and transferred to a PVDF membrane (Pierce Biotechnologies Inc., Rockford, IL, USA). The primary antibodies used in the study included anti-PPDPF (Proteintech, 19912-1-AP, 1:1000), anti-BABAM2 (Proteintech, 11702-1-AP, 1:1000), anti-HSP90 (Santa Cruz, sc-69703, 1:3000), anti-tubulin (Santa Cruz, sc-5286, 1:4000), anti-GAPDH (Santa Cruz, sc- 47724, 1:4000), anti-Caspase 3 (CST, 9662S, 1:1000), anti-Flag (Sigma, F1804; 1:3000), anti-ubiquitin(Proteintech, 10201-2-AP, 1:2000), anti-MYC (Proteintech, 16286-1-AP, 1:2000), anti-MDM2 (Santa Cruz, sc-965, 1:1000), anti-Bcl-xl (Santa Cruz, sc-8392, 1:1000), and anti-γH2AX (CST, 9718S, 1:2000). The signals were detected with HRP-conjugated secondary antibodies (Millipore).

### Immunoprecipitation (IP)

To detect the interaction between exogenous PPDPF (MYC-PPDPF) and BABAM2 (Flag-BABAM2), MYC-PPDPF and Flag-BABAM2 plasmids were transfected into HEK293T cells. Forty-eight hours after transfection, cells were lysed with IP lysis buffer (50 mM Tris-HCl (pH 8.0), 150 mM NaCl, 0.1% NP-40, and a protease and phosphatase inhibitor), and the supernatant was collected. Beads coupled to an anti-Flag antibody (Sigma, F1804) or an anti-MYC antibody (Proteintech, 16286-1-AP) were added to the supernatant and incubated for 4 hours. Then, the beads were washed 3 times with wash buffer (50 mM Tris-HCl (pH 8.0), 150 mM NaCl, and 0.1% NP-40), 1× loading buffer was added, and the beads were heated at 100 °C for 5 min. Then, the supernatant was collected for western blot analysis.

To detect any interaction between endogenously expressed PPDPF and BABAM2 in an NSCLC cell line, A549 cells were lysed with IP lysis buffer containing a protease and phosphatase inhibitor. Equal amounts of protein were aliquoted, and 0.2 μg of an anti-BABAM2 antibody was added and incubated overnight at 4 °C. The next day, 60 μl of Protein A/G beads (Bimake, B23202) was added and incubated overnight at 4 °C. The beads were washed 3 times with wash buffer, and 1× loading buffer was then added for western blot analysis.

### Yeast Two-hybridization

The DNA sequence encoding human PPDPF was subcloned into pGBKT7 (Clontech Laboratories) to yield a fusion protein with the Gal4 DNA-binding domain. This Gal4-PPDPF fusion protein was used as the bait for screening a human brain cDNA library (Clontech Laboratories) in pACT2 according to the manufacturer's instructions. Yeast transformation was performed in the strain AH109 with the polyethylene glycol-lithium acetate method, and positive clones were obtained by growing on selective media. DNA inserts from candidate clones were analyzed by sequencing.

### Cell Viability and Apoptosis Assays

Cell viability was evaluated by a CCK-8 assay (Bimake, B34302) following the manufacturer's guidelines. All viability experiments were repeated three times independently, and Student's *t* test was used to determine statistical significance. The apoptosis assay was carried out according to the apoptosis kit instructions (4A Biotech, FXP018-100).

### Soft agar colony formation assay

A double layer of agar was placed in a 24-well plate; the lower layer of agar was used to isolate cells from the plate bottom. A total of 8 × 10^2^ cells were mixed with the top agar and were then inoculated into the 24-well plate. Four weeks later, the nonanchored growth condition of cells was observed and photographed, and statistical analysis was then performed.

### Colony Formation Assay

NSCLC cells were seeded in 6-well plates at a density of 5 × 10^2^ cells per well. The next day, the cells were exposed to 0, 2, 4, 6, 8 and 10 Gy radiation. Two weeks later, the cells were washed three times with cold PBS and were then stained with 0.3% crystal violet (containing methanol). Colonies consisting of more than 50 cells were defined as surviving colonies. All viability measurements were normalized to the measurement in the corresponding 0 Gy group. Cell survival curves were fitted with GraphPad software.

### Transwell invasion assay

65 ul Matrigel diluted with basal RPMI-1640 medium (v:v,100:3) was added to the center of the bottom membrane in the upper chamber and incubated at 37 °C for 30 minutes to solidify. Cell suspension was prepared with a culture solution containing 0.1% FBS, and its density was adjusted to 5 × 10^5^ cells/ml. Then, 200 µL of the cell suspension was inoculated into the upper chambers, and 500 µL of medium containing 30% FBS was added to the lower chambers of the 24-well plate, and then plate was placed in an incubator at 37 °C in 5% CO_2_ for 60 hours. After washing three times with PBS, the chamber membranes were fixed with 4% paraformaldehyde and stained with 0.3% crystal violet. Five fields of view were randomly selected for observation by microscopy (Leica DMI4000B, Germany) at 20× magnification, and cells were then imaged and counted with ImageJ software (National Institutes of Health, Bethesda, MD, USA) to calculate the relative invasion rate.

### Immunohistochemistry

NSCLC tissue arrays were purchased from Shanghai OUTDO Biotech. The tissue arrays were deparaffinized and rehydrated prior to antigen retrieval and blocking of endogenous peroxidase activity. The sections were washed three times with 0.01 mol/L PBS (2 mmol/L NaH_2_PO4, 8 mmol/L Na_2_HPO4 and 150 mmol/L NaCl). Then, 0.01 mol/L PBS containing 5% normal goat serum and 0.1% Triton X-100 was used to block the sections, followed by incubation with an anti-PPDPF antibody (1:100; Proteintech, 19912-1-AP) overnight at 4 °C. After three washes in PBS and treatment with adjuvant for 20 min at 37 °C, the sections were incubated with a secondary antibody for 20 min at 37 °C. The immunohistochemical reaction was visualized with 3,3'-diaminobenzidine (DAB) for 2 min. All sections were counterstained with hematoxylin. Both the staining intensity and extent of protein expression were automatically scored with a Vectra 2 system (Perkin-Elmer, USA).

### Lung cancer mouse model and xenograft model

Kras^G12D^;LKB^f/f^ (KL) and Kras^G12D^;LKB^f/f^; PPDPF^f/f^ (KPL) model mice were housed under standard conditions on a 12:12-hour dark: light cycle. To induce lung cancer, we anesthetized 8-week-old mice with 2.5% tribromoethanol, ensuring that the mice were fully anesthetized and had no reaction to pain. Ad-Cre (adenovirus) virus (OBIO) was inoculated (1 × 10^9^ pfu per mouse) using the intranasal/orthotropic infection protocol as described 48. Eight weeks later, the lungs were harvested, and IHC analysis was carried out.

To establish the human NSCLC xenograft model, 4- to 6-week-old male nude mice were purchased from Hunan SJA Laboratory Animal Company. H1299 cells (5 × 10^6^) infected with viral control vector or shPPDPF were injected into the right (shPPDPF) and left (control vector) flanks of nude mice to induce NSCLC tumor formation, respectively.

### Statistical analysis

SPSS 20.0 software and GraphPad Prism (version 8.0) were used for statistical analysis. Flow cytometry data was analyzed using FlowJo10.01. The details of the statistical methods for every experiment are indicated in the corresponding figure legends, and the data are presented as the mean ± SD values. The data were analyzed by either t test or ANOVA. P values are presented as asterisks in figures: *, *P* < 0.05; **, *P* < 0.01; ***, *P* < 0.001; ****, *P* < 0.001.

## Supplementary Material

Supplementary figure and tables.

## Abbreviations

PPDPF: pancreatic progenitor cell differentiation and proliferation factor
NSCLC: non-small cell lung cancer)
BABAM2: BRISC and BRCA1-A complex member 2
DSB: Double-strand breaks
HR: Homologous recombination
NHEJ: Nonhomologous end joining
MDM2: Murine double minute 2
BRCA1: Breast Cancer Susceptibility Protein-1
BRCC: BRCA1-BRCA2-containing complex
XIAP: X-linked inhibitor of apoptosis
