# Supplementary material for: PPDPF Promotes the Progression and acts as an Antiapoptotic Protein in Non-Small Cell Lung Cancer
Source: Int J Biol Sci. 2022 Jan 1;18(1):214–28. doi: 10.7150/ijbs.65654 (PMC8692159; doi:10.7150/ijbs.65654)
Supplement: Supplementary file 1 — Supplementary figure and tables. [file ijbsv18p0214s1.pdf]

The Supplemental Materials Inventory

The Supplemental Materials contained 1 figures and 1 tables.

Supplemental Figures

Figure S1. PPDPF and BABAM2 led to radioresistance.

(A) PI-Annexin V staining was performed to examine the apoptosis of A549 cells with PPDPF knockdown. Cells were treated with radiation at a dose of 4 Gy. Twenty-four hours later, the cells were collected, and PI-Annexin V staining and FACS analysis was performed.

(B) PI-Annexin V staining was performed to examine the apoptosis of A549 cells overexpressing PPDPF. Cells were treated with radiation at a dose of 4 Gy. Twenty-four hours later, the cells were collected, and PI-Annexin V staining and FACS analysis was performed.

(C) PI-Annexin staining was performed to examine the rescue effects of BABAM2 overexpression on apoptosis induced by PPDPF knockdown. Cells were treated with radiation. Twenty-four hours later, PI-Annexin V staining and FACS analysis was performed.

Supplemental Table

Table S1 The information of patients tissues in Figure 1D.

Table S2 The information of patients in lung cancer tissue array.

Table S3 The Candidate E3 ligases.

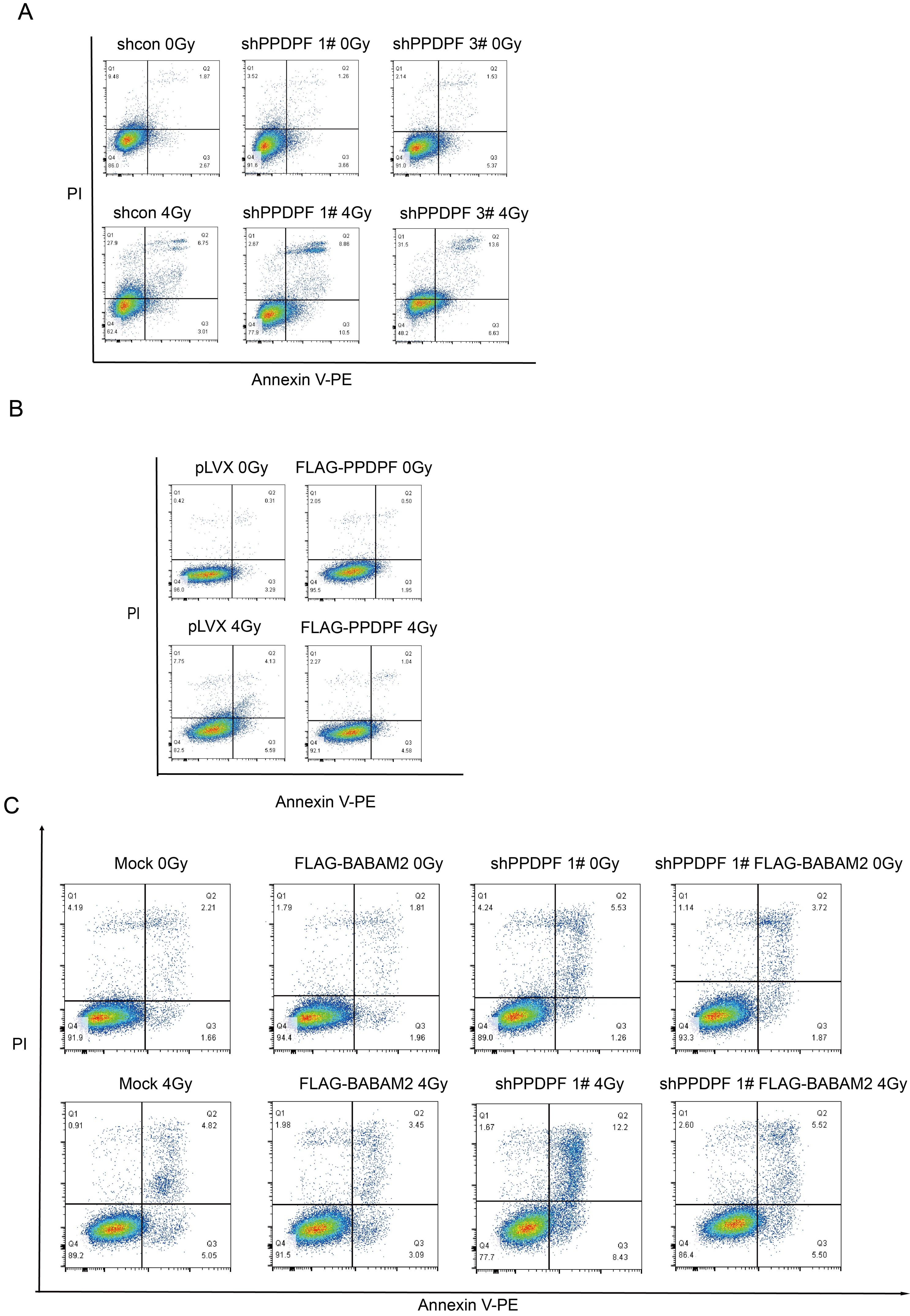

A
shcon 0Gy
shPPDPF 1# 0Gy
shPPDPF 3# 0Gy
shcon 4Gy
shPPDPF 1# 4Gy
shPPDPF 3# 4Gy
PI
Annexin V-PE
B
pLVX 0Gy
FLAG-PPDPF 0Gy
pLVX 4Gy
FLAG-PPDPF 4Gy
PI
Annexin V-PE
C
Mock 0Gy
FLAG-BABAM2 0Gy
shPPDPF 1# 0Gy
shPPDPF 1# FLAG-BABAM2 0Gy
Mock 4Gy
FLAG-BABAM2 4Gy
shPPDPF 1# 4Gy
shPPDPF 1# FLAG-BABAM2 4Gy
PI
Annexin V-PE

Table S1. The information of patients tissues in Figure 1D.

	Age	Gender	Surgery date	Vital Status	Tobacco Smoking History	Family History of Lung Cancer	ECOG Score	Tumor Site	Tumor _Size _in_cm	Histologic Type	Histologic Grade
Case 1	62	Female	2018/3/12	Alive	No	No	0	Lower Lobe	2.4	Adenocarcinoma	Middle Differentiation
Case 2	70	Male	2018/5/21	Alive	Yes	No	0	Upper Lobe	5.2	Squamous Cell Carcinoma	High Differentiation
Case 3	58	Male	2018/5/25	Alive	Yes	Father	0	Upper Lobe	4.1	Adenocarcinoma	Middle Differentiation
Case 4	79	Female	2018/5/28	Alive	No	No	0	Upper Lobe	3.3	Adenocarcinoma	High-Middle Differentiation
Case 5	72	Female	2018/5/31	Alive	No	No	0	Lower Lobe	2.4	Adenocarcinoma	Middle Differentiation
Case 6	67	Male	2018/6/4	Alive	No	No	0	Lower Lobe	3.4	Adenocarcinoma	High-Middle Differentiation
Case 7	64	Female	2018/6/4	Alive	No	No	0	Upper Lobe	3.1	Adenocarcinoma	High-Middle Differentiation
Case 8	35	Female	2018/6/21	Alive	No	Brother	0	Upper Lobe	2.4	Adenocarcinoma	Middle Differentiation
Case 9	80	Male	2018/7/30	Alive	Yes	No	0	Upper Lobe	4	Adenocarcinoma	Middle Differentiation
Case 10	61	Male	2018/8/1	Alive	Yes	NO	0	Lower Lobe	3.7	Adenocarcinoma	Medium-Low Differentiation

Case 11	71	Male	2018/9/5	Alive	No	NO	0	Upper Lobe	2.8	Adenocarcinoma	Medium-Low Differentiation
Case 12	66	Male	2018/9/5	Alive	Yes	NO	0	Lower Lobe	5.5	Squamous Cell Carcinoma	Keratinizing

Table S2. The information of patients in lung cancer tissue array

	Surgery date	Vital Status	Overall survival(up to 2016.6)	Tissue code	Gender	Age	Distant Metastasis Site	Primary Tumor	Histologic Type	Tumor_Stage_Pathological
Tumor/Adjacent	2008/1/15	living	50	E05A3073	Male	35	No	Yes	Adenocarcinoma	II-III
Tumor/Adjacent	2008/2/26	living	24	E05A3146	Male	65	No	Yes	Adenocarcinoma	II
Tumor/Adjacent	2008/4/7	living	55	E05A2666	Female	62	No	Yes	Adenocarcinoma	II
Tumor/Adjacent	2008/7/14	dead	16	E05A1973	Male	49	No	Yes	Adenocarcinoma	II
Tumor/Adjacent	2008/7/15	dead	42	E05A2597	Female	50	No	Yes	Adenocarcinoma	II
Tumor/Adjacent	2009/4/14	dead	22	E05A2605	Male	74	No	Yes	Adenocarcinoma	III
Tumor/Adjacent	2009/6/17	living	54	E05A2707	Male	62	No	Yes	Adenocarcinoma	II
Tumor/Adjacent	2009/6/19	dead	20	E05A1303	Male	70	No	Yes	Adenocarcinoma	II-III
Tumor/Adjacent	2009/7/13	dead	9	E05A1104	Male	53	No	Yes	Adenocarcinoma	III
Tumor/Adjacent	2009/7/14	dead	15	E05A3066	Female	69	No	Yes	Adenocarcinoma	II-III
Tumor/Adjacent	2009/7/31	dead	8	E05A1974	Male	60	No	Yes	Adenocarcinoma	III
Tumor/Adjacent	2009/7/30	living	56	E05A2660	Female	51	No	Yes	Adenocarcinoma，partial squamous cell carcinoma	II-III
Tumor/Adjacent	2009/9/28	living	73	E05A1118	Male	54	No	Yes	Adenocarcinoma，partial squamous cell carcinoma	I-II
Tumor/Adjacent	2009/10/29	dead	23	E05A3074	Male	60	Nodules of chest wall carcinoma	Yes	Adenocarcinoma	II-III
Tumor/Adjacent	2009/10/5	dead	5	E05A1031	Male	69	No	Yes	Adenocarcinoma	III
Tumor/Adjacent	2009/10/26	dead	9	E05A2132	Male	70	No	Yes	Adenocarcinoma	II

Tumor/Adjacent	2009/11/3	living	73	E05A1120	Female	68	No	Yes	Adenocarcinoma	II-III
Tumor/Adjacent	2009/12/5	living	24	E05A3144	Female	60	No	Yes	Papillary Adenocarcinoma	I-III
Tumor/Adjacent	2009/12/15	dead	11	E05A0749	Female	56	No	Yes	Adenocarcinoma	II
Tumor/Adjacent	2009/12/15	living	62	E05A2143	Male	61	No	Yes	Adenocarcinoma	II-III
Tumor/Adjacent	2009/12/25	dead	32	E05A1811	Female	62	No	Yes	Adenocarcinoma，partial bronchioloalveolar carcinoma	II
Tumor/Adjacent	2010/1/5	dead	41	E05A3172	Male	69	No	Yes	Adenocarcinoma，partial papillary	II
Tumor/Adjacent	2010/1/14	living	62	E05A2142	Male	59	No	Yes	Adenocarcinoma	III
Tumor/Adjacent	2010/4/26	dead	46	E05A3071	Female	68	No	Yes	Adenocarcinoma	II
Tumor/Adjacent	2010/5/11	living	28	E05A3129	Male	75	No	Yes	Mixed Adenocarcinoma	I-III
Tumor/Adjacent	2010/5/25	dead	39	E05A1028	Male	52	No	Yes	Adenocarcinoma	II
Tumor/Adjacent	2010/6/9	dead	13	E05A3079	Female	63	No	Yes	Adenocarcinoma	II
Tumor/Adjacent	2010/6/11	dead	25	E05A1051	Female	71	No	Yes	Adenocarcinoma	II
Tumor/Adjacent	2010/7/15	dead	12	E05A1132	Male	57	No	Yes	Adenocarcinoma	II-III
Tumor/Adjacent	2010/7/21	living	46	E05A3114	Female	80	No	Yes	Adenocarcinoma	II
Tumor/Adjacent	2010/7/30	dead	31	E05A2825	Female	75	No	Yes	Adenocarcinoma	II
tumor	2010/8/23	dead	38	E05A2138	Male	65	No	Yes	Adenocarcinoma，partial Large cell neuroendocrine carcinoma	II
Tumor/Adjacent	2010/9/13	dead	83	E05A0777	Male	76	No	Yes	Adenocarcinoma	I-II
Tumor/Adjacent	2010/9/13	living	57	E05A2657	Male	62	No	Yes	Adenocarcinoma	II
Tumor/Adjacent	2010/10/28	living	43	E05A3125	Female	44	No	Yes	Adenocarcinoma	II-III
Tumor/Adjacent	2010/11/2	dead	21	E05A3117	Female	57	No	Yes	Adenocarcinoma	II
Tumor/Adjacent	2010/11/25	living	83	E05A0772	Female	60	No	Yes	Adenocarcinoma，partial Signet-ring cell carcinoma	III

Tumor/Adjacent	2010/12/9	dead	42	E05A1810	Male	54	No	Yes	Adenocarcinoma，partial mucinous Adenocarcinoma	II-III
Tumor/Adjacent	2010/12/16	living	49	E05A3077	Female	64	No	Yes	Adenocarcinoma	II
Tumor/Adjacent	2010/12/21	dead	7	E05A2668	Male	79	No	Yes	Adenocarcinoma	II
Tumor/Adjacent	2010/12/31	dead	39	E05A2263	Female	66	No	Yes	Adenocarcinoma	II-III
Tumor/Adjacent	2011/1/12	dead	33	E05A0997	Male	57	Brain	Yes	Adenocarcinoma	II
Tumor/Adjacent	2011/1/13	dead	17	E05A3392	Male	63	No	Yes	Adenocarcinoma	II-III
Tumor/Adjacent	2011/1/19	dead	28	E05A3099	Female	78	No	Yes	Adenocarcinoma	II
Tumor/Adjacent	2011/2/1	dead	46	E05A1937	Male	63	No	Yes	Adenocarcinoma	I-II
Tumor/Adjacent	2011/3/16	dead	9	E05A3393	Male	65	No	Yes	Adenocarcinoma，partial large cell carcinoma	II-III
Tumor/Adjacent	2011/4/21	living	71	E05A1795	Male	73	No	Yes	Adenocarcinoma	I-II
Tumor/Adjacent	2011/4/22	dead	27	E05A1931	Male	52	No	Yes	Adenocarcinoma，partial mucinous Adenocarcinoma	II-III
Tumor/Adjacent	2011/6/17	living	67	E05A1823	Female	73	No	Yes	Adenocarcinoma，partial bronchioloalveolar carcinoma	II-III
Tumor/Adjacent	2011/8/5	dead	47	E05A1808	Female	56	No	Yes	Adenocarcinoma，partial mucinous Adenocarcinoma	II
Tumor/Adjacent	2011/8/9	dead	12	E05A2702	Female	83	No	Yes	Adenocarcinoma	II-III
Tumor/Adjacent	2011/8/9	dead	9	E05A1046	Male	76	No	Yes	Adenocarcinoma	II-III
Tumor/Adjacent	2011/8/24	dead	40	E05A0773	Female	68	No	Yes	Adenocarcinoma	II-III
Tumor/Adjacent	2011/9/15	living	66	E05A1933	Female	55	No	Yes	Adenocarcinoma	II
Tumor/Adjacent	2011/10/11	dead	34	E05A3063	Male	76	No	Yes	Adenocarcinoma	II-III
Tumor/Adjacent	2011/10/13	living	72	E05A1134	Female	61	No	Yes	Adenocarcinoma	II
Tumor	2011/11/1	living	27	E05A3140	Male	75	No	Yes	Adenocarcinoma	I

Tumor/Adjacent	2011/11/9	dead	16	E05A0809	Male	72	No	Yes	Adenocarcinoma	II
Tumor/Adjacent	2011/11/18	dead	21	E05A3058	Female	54	No	Yes	Adenocarcinoma	II
Tumor/Adjacent	2011/11/28	living	42	E05A3168	Female	40	No	Yes	Adenocarcinoma	II
Tumor/Adjacent	2011/12/1	living	48	E05A3094	Female	52	No	Yes	Adenocarcinoma	I-III
Tumor/Adjacent	2011/12/21	living	58	E05A2598	Male	44	No	Yes	Adenocarcinoma	II
Tumor/Adjacent	2011/12/20	dead	22	E05A1930	Male	73	No	Yes	Adenocarcinoma	II
Tumor/Adjacent	2012/1/19	living	70	E05A1800	Female	69	No	Yes	Adenocarcinoma	II
Tumor/Adjacent	2012/3/8	dead	27	E05A2704	Male	63	No	Yes	Adenocarcinoma	II
Tumor/Adjacent	2012/3/14	living	64	E05A1977	Male	54	No	Yes	Adenocarcinoma	I-II
Tumor/Adjacent	2012/3/22	dead	47	E05A3085	Male	53	No	Yes	Adenocarcinoma，partial Signet-ring cell Adenocarcinoma	III
Tumor/Adjacent	2012/3/27	living	48	E05A3088	Female	65	No	Yes	Adenocarcinoma	II
Tumor/Adjacent	2012/3/31	living	48	E05A3087	Male	59	No	Yes	Adenocarcinoma	II
Tumor/Adjacent	2012/4/9	dead	4	E05A2599	Female	52	No	Yes	Adenocarcinoma	II
Tumor/Adjacent	2012/5/3	dead	43	E05A0779	Male	65	No	Yes	Adenocarcinoma	II
Tumor/Adjacent	2012/5/7	living	65	E05A2528	Male	53	No	Yes	Adenocarcinoma	II
Tumor/Adjacent	2012/5/17	dead	14	E05A2658	Male	60	No	Yes	Adenocarcinoma，with Neuroendocrine change	II
Tumor/Adjacent	2012/5/28	living	43	E05A3173	Female	55	No	Yes	Adenocarcinoma	II
Tumor/Adjacent	2012/6/6	dead	46	E05A0951	Female	53	No	Yes	Adenocarcinoma	I-III
Tumor/Adjacent	2012/6/13	living	54	E05A2827	Female	66	No	Yes	Adenocarcinoma	II
Tumor/Adjacent	2012/6/13	living	42	E05A3179	Male	67	No	Yes	Adenocarcinoma，with sarcomatous change	II-III

Tumor/Adjacent	2012/6/26	dead	4	E05A1027	Male	77	No	Yes	Adenocarcinoma	II-III
Tumor/Adjacent	2012/7/20	dead	5	E05A1301	Male	86	No	Yes	Adenocarcinoma，partial squamous cell carcinoma	II
Tumor	2012/8/31	dead	14	E05A1746	Male	78	No	Yes	Adenocarcinoma, partial adenosquamous carcinoma	II-III
Tumor/Adjacent	2012/9/14	living	8	E05A1738	Female	76	No	Yes	bronchioloalveolar carcinoma	II
Tumor/Adjacent	2012/11/2	living	59	E05A1708	Female	73	No	Yes	Adenocarcinoma	II
Tumor/Adjacent	2012/11/12	living	57	E05A1722	Female	73	No	Yes	Adenocarcinoma	II
Tumor/Adjacent	2012/11/16	living	53	E05A1740	Male	66	No	Yes	Adenocarcinoma，partial sarcomatoid carcinoma	II
Tumor	2012/12/4	living	69	E05A1650	Male	64	No	Yes	Adenocarcinoma	II
Tumor/Adjacent	2012/12/31	dead	33	E05A1766	Female	64	No	Yes	Adenocarcinoma	II-III
Tumor/Adjacent	2013/7/18	dead	35	E05A1700	Female	63	No	Yes	Adenocarcinoma	II
Tumor/Adjacent	2013/7/23	living	51	E05A1750	Male	59	No	Yes	Adenocarcinoma	II-III
Tumor/Adjacent	2008/6/18	living	74	E05A1605	Female	57	No	Yes	bronchioloalveolar carcinoma	I-II
Tumor/Adjacent	2009/11/19	dead	20	E05A1659	Female	55	No	Yes	Adenocarcinoma	II
Tumor/Adjacent	2011/4/6	living	68	E05A1662	Male	53	No	Yes	bronchioloalveolar carcinoma	II
Tumor/Adjacent	2011/12/29	living	71	E05A1636	Male	51	No	Yes	bronchioloalveolar carcinoma	II

Table S1. **The Candidate E3 ligases.**

E3	E3GENE	SUBGENE	HOMO	PFAM	GO	NET	MOTIF	SCORE
P46934	NEDD4	BRE	1	1	2.88	1.29	8.67	0.819
Q96J02	ITCH	BRE	1	1	1.24	1.29	8.67	0.758
Q969H0	FBXW7	BRE	1	1	1.13	1.29	8.67	0.751
Q00987	MDM2	BRE	1	1	2.88	1.44	2.8	0.744
Q86TM6	SYVN1	BRE	1	1	1.25	1	8.67	0.738
O43164	PJA2	BRE	1	1	1.51	1	6.61	0.731
Q86YT6	MIB1	BRE	1	1	1.51	1	6.61	0.731
Q96AX9	MIB2	BRE	1	1	1.13	1	6.61	0.705
Q13309	SKP2	BRE	1	1	2.33	1	2.12	0.667
Q9UNE7	STUB1	BRE	1	1	3.77	1.29	1	0.665
Q9HCE7	SMURF1	BRE	1	1	4.05	1	1.06	0.653
P22681	CBL	BRE	1	1	2.88	1.29	1.06	0.645
Q5T0T0	MARCH8	BRE	1	1	1.13	1	3.41	0.642
P38398	BRCA1	BRE	1	1	2.93	1.68	1	0.641
Q9NXR7	BRE	BRE	1	1	2.93	1.3	1	0.641
P35226	BMI1	BRE	1	1	2.93	1.29	1	0.64
P46736	BRCC3	BRE	1	1	2.93	1.29	1	0.64
O76064	RNF8	BRE	1	1	2.93	1.29	1	0.64
P29590	PML	BRE	1	1	2.88	1.29	1	0.639
Q86Y01	DTX1	BRE	1	1	2.88	1.29	1	0.639
Q9UKV5	AMFR	BRE	1	1	1.25	1	2.8	0.633
Q96Q27	ASB2	BRE	1	1	1	1	3.41	0.63
Q09472	EP300	BRE	1	1	2.33	1.44	1	0.628
Q9UM11	FZR1	BRE	1	1	1.51	1	2.12	0.624
Q9UH77	KLHL3	BRE	1	1	1.13	1	2.8	0.623
Q8TCQ1	MARCH1	BRE	1	1	1.13	1	2.8	0.623
Q86YJ5	MARCH9	BRE	1	1	1.13	1	2.8	0.623
P02511	CRYAB	BRE	1	1	2.33	1.29	1	0.617
P51948	MNAT1	BRE	1	1	2.33	1.29	1	0.617
Q99728	BARD1	BRE	1	1	2.33	1.29	1	0.617
Q9ULJ6	ZMIZ1	BRE	1	1	2.93	1	1	0.615
Q9UDY8	MALT1	BRE	1	1	2.93	1	1	0.615
Q9UPN9	TRIM33	BRE	1	1	2.93	1	1	0.615
Q969V5	MUL1	BRE	1	1	2.93	1	1	0.615
P78406	RAE1	BRE	1	1	2.93	1	1	0.615
Q8IYW5	RNF168	BRE	1	1	2.93	1	1	0.615
O14512	SOCS7	BRE	1	1	2.88	1	1	0.613
O60315	ZEB2	BRE	1	1	2.88	1	1	0.613
Q9ULV8	CBLC	BRE	1	1	2.88	1	1	0.613
Q13216	ERCC8	BRE	1	1	2.88	1	1	0.613
Q8WWQ0	PHIP	BRE	1	1	2.88	1	1	0.613

P62873	GNB1	BRE	1	1	2.88	1	1	0.613
Q9P1Y6	PHRF1	BRE	1	1	2.88	1	1	0.613
P43034	PAFAH1B1	BRE	1	1	2.88	1	1	0.613
Q15386	UBE3C	BRE	1	1	1.25	1	2.12	0.604
